# Sowing Methods Influence Soil Bacterial Diversity and Community Composition in a Winter Wheat-Summer Maize Rotation System on the Loess Plateau

**DOI:** 10.3389/fmicb.2020.00192

**Published:** 2020-02-18

**Authors:** Chunguo Huang, Xiaoli Han, Zhenping Yang, Yinglong Chen, Zed Rengel

**Affiliations:** ^1^College of Agriculture, Shanxi Agricultural University, Jinzhong, China; ^2^College of Forestry, Shanxi Agricultural University, Jinzhong, China; ^3^Institute of Agriculture, UWA School of Agriculture and Environment, The University of Western Australia, Perth, WA, Australia; ^4^Institute of Soil and Water Conservation, Chinese Academy of Sciences, Northwest A&F University, Yangling, China

**Keywords:** bacterial community composition, diversity, sowing methods, rhizosphere, soil layers

## Abstract

Soil bacterial diversity and community composition are crucial for soil health and plant growth, and their dynamics in response to agronomic practices are poorly understood. The aim of this study was to investigate the response of soil bacterial community structure to the changes of sowing methods, soil depth and distance to roots in a winter wheat-summer maize crop rotation system on the Loess Plateau in china (35°17′38′′N, 111°40′24′′E). The experiment was laid out as completely randomized block design with three replications. Sowing methods trialed were: traditional sowing (TS), film-mulched ridge and furrow sowing (FMR&F), wide ridge and narrow furrow sowing (WR&NF) and unplanted control (CK). The result showed that the WR&NF sowing method treatment significantly decreased soil bacterial diversity (Chao 1 and Shannon indices) compared to the TS and FMR&F treatment, but increased abundance of beneficial bacteria such as genera *Bacillus* and *Pseudomonas* compared to the TS treatment. These genera showed a stronger correlation with soil properties and contributed to the soil nutrient cycling and crop productivity. *Bacillus, Pseudomonas, Nevskia*, *a*nd *Lactococcus* were the keystone genera in this winter wheat-summer maize rotation system on the Loess Plateau. Strong correlations between changes in soil properties and soil bacterial diversity and abundance were identified. In summary, we suggest that the WR&NF treatment, as a no-mulching film and no-deep tillage sowing method, would be the most suitable sowing technique in the winter wheat-summer maize rotation on Loess soil.

## Introduction

Soil is a complex and dynamic system responsible for crop growth and development. Soil microbial communities are the key factor in providing ecosystem functions (Sengupta and Dick, 2015). Soil processes are mediated by microorganisms (including maintenance of soil structure, organic matter decomposition, and nutrient recycling) influencing the above-ground plant growth and productivity (Glick, 1995; Nacke et al., 2011; Nannipieri et al., 2017). In particular, soil microbial activity has been reported as an important component of soil function in organic matter mineralization to provide nitrogen, phosphorus and potassium in agricultural ecosystems (Grayston et al., 1998; Simmons and Coleman, 2008).

The spatial distribution of soil microbial diversity and abundance, such as in rhizosphere versus bulk soil, or down the soil profile, have gained much attention because of soil fertility concerns. These microbial communities are influenced by the plant root system (Chen et al., 2007), soil pH (Lauber et al., 2009) and organic matter availability (Wakelin et al., 2008). Soil microbial parameters (e.g., diversity and abundance) are important indicators of soil quality (Doran and Zeiss, 2000), that are influenced strongly by the land management practices (Buckley and Schmidt, 2003) and seasonal changes (Lipson and Schmidt, 2004). Soil physical disturbance alters soil micro-environment due to changes in soil properties (including soil moisture, temperature, aeration, organic matter stratification, nutrient distribution, etc.), which further influence soil micro-aggregation and soil microbial communities (Kladivko, 2001; Lienhard et al., 2013; Sengupta and Dick, 2015).

Conventional farming techniques, where crop residues are incorporated into soil, are prevalent on Loess Plateau of China. Such incorporation can boost crop residue decomposition and soil microbiome structure by relocating food resources and exposing protected carbon (Beare et al., 1992; Moore, 1994; Hartmann et al., 2015); in turn, microbial abundance, activity and diversity can influence the sustainable productivity of farming systems (Van Der Heijden et al., 2008). The previous research has focused on the soil microbial diversity and community composition under different tillage treatment and fertilization regimes (Mijangos et al., 2006). It has been argued that bacterial community composition and diversity differ with soil depth and across the rhizosphere/non-rhizosphere boundary in different crops.

Traditional sowing method (TS, drilled using a mechanical seeder, with rows spaced 20 cm apart without film mulching), is widely practiced on Loess Plateau in China. Such sowing method without mulching does not conserve precipitation and soil moisture (Liu et al., 2005). Several alternatives, such as the film-mulched ridge and furrow sowing method (FMR&F), with film-mulched ridges (an arc with 40 cm wide base and 10 cm height) and seeding into the furrow (rows spaced 15 cm) by using an all-in-one machine, combining ridging, mulching, fertilization, and sowing, are arising to replace the TS method. However, plastic film recycling is an issue (Liu et al., 2009; Li et al., 2018). The wide ridge and narrow furrow sowing method (WR&NF), with wide ridge (25 cm wide base and 12 cm height) and narrow furrow (depth 8 cm, sown into the top-edges of the furrow, rows spaced 12 cm) by using an all-in-one machine for ridging, fertilization and sowing, is being promoted not only for conserving precipitation and decreasing soil water evaporation, but also for avoiding contamination of soil environment with plastic (Sun et al., 2015). Li et al. (2018) reported that various sowing methods influenced wheat yield due to changes in soil water storage and water-use efficiency on the Loess Plateau in China. However, it remained unclear how different sowing methods would influence soil bacterial diversity and abundance that contribute to the changes in soil quality and micro-environment (Mann et al., 2019).

In this study, we applied Illumina HiSeq sequencing analysis of the V3-V4 16S rRNA gene region to characterize soil bacterial diversity and abundance among different soil layers down the profile, and between rhizosphere and non-rhizosphere soil as influenced by various sowing methods. We hypothesize that (a) soil bacteria diversity and community structure decrease by some sowing methods, which influence soil physicochemical properties; (b) the wide ridge and narrow furrow sowing method (WR&NF) treatments increase the abundance of beneficial bacterial genera, in particular bio-control bacterial taxa; (c) bacterial diversity decrease down the soil profile; and (d) the rhizosphere niche has lower abundance of predominant taxa than the non-rhizosphere environment.

## Materials and Methods

### Experimental Design and Soil Sampling

The experiment was conducted in the field (35°17′38′′N, 111°40′24′′E) with long-term winter wheat-summer maize rotation systems, in Yuanqu County, Shanxi Province, located on the Loess Plateau in the northwest of China. This region has a sub-humid, warm, temperate, continental monsoon climate. The annual temperature is 13.5°C and annual precipitation is 631 mm, with 230 frost-free days. The soil is medium loam and classified as cinnamon red vertical structural Loess based on the Chinese Soil Taxonomy (Xue et al., 2018; Zhang et al., 2018). The soil properties of cultivated horizon (0–20 cm) were 10.5 g kg^–1^ of soil organic matter (SOM), 0.71 g kg^–1^ of total nitrogen, 86 mg kg^–1^ of available N, 14.5 mg kg^–1^ of available phosphorus and 118.2 mg kg^–1^ of available potassium in October 10, 2014 before sowing winter wheat.

In this experiment, the summer maize was sown on June 20, 2014 and harvested on October 7, 2014. The following winter wheat crop was sown on October 13, 2014 and harvested on June 14, 2015. On the day of harvest, we machine-shattered maize stem residues (pieces < 5 cm in length) and mixed them with the top 30 cm of soil. The field experiment in the completely randomized block design was laid out using winter wheat (*Triticum aestivum* L., variety Yannong 21) with three replications. Four treatments were implemented as the sowing methods: (A) traditional sowing method (TS) with rows spaced 20 cm apart without film mulching and furrowing, (B) film-mulched ridge and furrow sowing method (FMR&F) with film-mulched ridge (an arc with 40 cm wide base and 10 cm height and film-mulched with 0.01-mm-thick polyethylene) and seeding into the furrow (rows spaced 15 cm), (C) wide ridge and narrow furrow sowing method (WR&NF), with wide ridges (with 25 cm wide base and 12 cm height) and narrow furrow (depth 8 cm, sown into the top edges of the furrow, rows spaced 12 cm), and (D) control (CK) plots left unplanted (Supplementary Figure S1). The plot size was 0.133 ha, with a seeding rate of 112.5 kg ha^–1^. Approximately 750 kg ha^–1^ N-P-K (18-22-5%) compound fertilizer (equivalent to 135 kg N ha^–1^, 165 kg P_2_O_5_ ha^–1^, and 37.5 kg K_2_O ha^–1^) was applied as a basal fertilizer to all plot at the time of sowing winter wheat. All treatments were implemented by using an All-in-One Seedling Machine (Taicang Xiangshi Agricultural Machinery Co., Ltd., China) for ridging or furrowing, fertilization and seeding. No irrigation was applied during the two crops season. All experimental plots received the same management based on the standard recommended practices for growing wheat in the region.

After winter wheat was harvested on June 14, 2015, five soil cores were collected in each plot (in 20 cm increments from 0 to 60 cm depth, respectively) using a soil-drilling sampler (10 cm inner diameter). Five cores from the same depth and the same plot were combined to form three mixed samples for respective soil depths i.e., 0–20, 20–40, and 40–60 cm (Gregorich and Carter, 2007). Each sample was divided into rhizosphere soil (R) (dislodged from roots by using sterilized toothpicks) and non-rhizosphere soil (N) (obtained by gently shaking roots), except in the unplanted control plot; there were sixty-three soil samples in total (Gil et al., 2011). All soil samples were sieved through a 2- mm mesh, and each soil sample was then divided into two halves. One half was placed in a 50-mL centrifuge tube with added liquid N. The tubes stored in a dry-ice box were transported to the laboratory immediately, and kept at −80°C until DNA extraction. Another half of the same soil sample was used for measurement of soil physicochemical properties in a laboratory.

### Measurement of Soil Physicochemical Properties

Soil organic matter was determined by the chromic acid titration method (Ryan et al., 2007). Soil moisture content was calculated from three homogenized replicates. After oven drying and milling, hydrolysable nitrogen (N), available phosphorus (P), and available potassium (K) contents were quantified using Conway method, MADAC and flame photometry, respectively (Halstead and Chairen, 1950; Lauber et al., 2008; Guo et al., 2011; Cao et al., 2012; Chaudhari et al., 2013). Soil pH was measured from supernatant of 0.01 M CaCl_2_ soil slurries, 1:1 (w/v), after 10 min of vigorous shaking and soil particle settling (Thomas, 1996).

### DNA Extraction, PCR and Illumina HiSeq 2500 Sequencing

Total bacterial genomic DNA was extracted from 0.5 g of soil sample using the Fast DNA SPIN extraction kits (MP Biomedical, Santa Ana, CA, United States) based on the manufacturer’s protocol (Davinic et al., 2012). Using a NanoDrop ND-1000 spectrophotometer (Thermo Fisher Scientific, Waltham, MA, United States) and agarose gel electrophoresis, we assessed the quantity and quality of extracted DNA, respectively. The forward primer 338F (5′-ACTCCTACGGGAGGCAGCA-3′) and the reverse primer 806R (5′-GGACTACHVGGGTWTCTAAT-3′) were used for the PCR amplification of the bacterial 16S rRNA gene V3-V4 region (Langille et al., 2013). The sample-specific 7-bp barcodes were incorporated into the primers for multiplex sequencing. The PCR components contained 5 μL of Q5 reaction buffer (5×), 5 μL of Q5 High-Fidelity GC buffer (5×), 0.25 μL of Q5 High-Fidelity DNA Polymerase (5 U/μL), 2 μL of 2.5 mM dNTPs, 1 μL (10 μM) of each forward and reverse primers, 2 μL of DNA template, and 8.75 μL of ddH_2_O. The thermal cycling program was run as follows: initial denaturation at 98°C for 2 min, followed by 25 cycles consisting of denaturation at 98°C for 15 s, annealing at 55°C for 30 s, and extension at 72°C for 30 s, with a final extension of 5 min at 72°C. PCR amplicons were purified using AgencourtAMPure Beads (Beckman Coulter, Indianapolis, IN, United States) and quantified by a PicoGreen dsDNA Assay Kit (Invitrogen, Carlsbad, CA, United States). After the individual quantification step, amplicons were pooled in equal amounts, and the pair-end 2 × 300 bp sequencing was performed using an Illumina HiSeq platform with a HiSeq × Five Reagent Kit v2.5 at Shanghai Personal Biotechnology Co., Ltd (Shanghai, China).

### Data Processing and Bioinformatics Analyses

The raw FASTQ files were demultiplexed and quality-filtered using the Quantitative Insight Into Microbial Ecology (QIIME, v1.8.0)^1^ pipeline as described previously (Caporaso et al., 2010). The raw sequencing reads were assigned to respective samples and identified as valid sequences with the following criteria (Gill et al., 2006; Chen and Jiang, 2014): reads shorter than 150 bp and average Phred scores of <20 were discarded; reads with exact barcode matching, two nucleotide mismatches in primer matching or containing ambiguous bases, and mononucleotide repeats of >8 bp were removed. Paired-end reads were assembled using FLASH (v1.2.7)^2^ (Magoč and Salzberg, 2011). The remaining high-quality sequences were submitted to the SRA (Sequence Read Archive) at the National Center for Biotechnology Information (NCBI) under accession number SRP231783 for 16S sequences. Operational taxonomic units (OTUs) were clustered at 97% sequence identity by UCLUST (Edgar, 2010). Chimeric sequences were identified in each OTU using the UCHIME algorithm. OTU taxonomic classification was conducted by BLAST searching of the representative sequences set against the Green Genes databases (DeSantis et al., 2006) using the best hit (Altschul et al., 1997). An OTU table, Biological Observation Matrix (BIOM), was further generated to record the abundance of each OTU in each sample and the taxonomy of these OTUs. OTUs containing less than 0.001% of total sequences across all samples were discarded (Bokulich et al., 2013). On average, 71,688 high-quality 16S sequences were obtained per sample. To minimize the differences in sequencing depth across samples, a rarefied OTU table was generated using an average of 100 evenly resembled OTU subsets with the 90% minimum sequencing depth for further analysis. To normalize the data, a subset of the 64,519 high-quality sequences per sample were selected randomly using the Mothur software (v1.31.2)^3^. The rarefaction curves showed that the sequences data were representative of most samples (Supplementary Figure S2).

#### Calculations and Statistical Analysis

Sequence data analyses were mainly performed using QIIME and R packages (v3.2.0). OTU-level alpha-diversity indices, such as Chao1 richness estimator and Shannon diversity index were calculated using the OTU table in QIIME. Taxa abundances at the phylum and genus levels were statistically compared among samples or groups by Metastats (White et al., 2009). The generalization error was estimated using 10-fold cross-validation. The expected “baseline” error was also included, which was obtained by a classifier that simply predicts the most common category label. Co-occurrence analysis was performed by calculating Spearman’s rank correlations between the dominant taxa. Correlations with | ρ| > 0.8 and *p* < 0.01 were investigated via co-occurrence network using Cytoscape (Shannon et al., 2003).

Data analysis was performed in SPSS statistics 20.0 software using the general linear model to test for significant differences. SOM, available P, pH, bacterial diversity and predominant taxa were analyzed by one-way ANOVA. Soil moisture content was analyzed by two-way ANOVA with rhizosphere/non-rhizosphere and soil layers factors. Abundance of Firmicutes, *Bacillus* and *Pseudomonas* were analyzed by two-way ANOVA with the sowing method treatments and the soil layers factors. Orthogonal contrasts analysis and linear regression analysis were used to assess soil properties as influenced by sowing methods, rhizosphere/bulk soil and soil layers. Simple correlation coefficients of soil properties and diversity indices were calculated using the Pearson correlation analysis. Spearman’s rank correlations were used to correlate the dominant taxonomy and soil properties.

## Results

### Influence of Sowing Methods on Soil Properties and Bacterial Community Composition

Organic matter, available P and soil moisture content differed significantly in various sowing treatments, but no sowing treatment differences were noted in soil pH, available N and K (Table 1). The FMR&F treatment had higher organic matter content than the other three treatments that did not differ among themselves. The same treatment had higher available P and soil moisture content than the TS and WR&NF treatments.

**TABLE 1:** Physicochemical soil properties (1-way ANOVA).

Treatments	Organic matter (g/kg)	Available P (mg/kg)	pH_*water*_	Soil moisture (% w/w)	Available N (mg/kg)	Available K (mg/kg)
**Sowing methods**						
TS	8.9b	6.52c	7.12	29.8c	63	103
FMR&F	11.2a	8.12a	7.11	37.2a	63	103
WR&NF	9.4b	7.20bc	7.09	30.7bc	61	103
CK	9.6b	7.65ab	7.02	27.8d	66	105
**Sampling sites**						
rhizosphere	9.5b	6.98b	7.05b	34.1a	59 b	99 b
non-rhizosphere	10.0a	7.60a	7.12a	30.2b	66 a	106 a
**Soil layers**						
0–20 cm	13.8a	11.98a	7.17a	36.0a	94 a	129 a
20–40 cm	8.6b	5.97b	7.08ab	31.9b	53 b	95 b
40–60 cm	6.9c	4.04c	7.02b	27.7c	42 c	85 c

*a, b, c Means are significantly different at *p* ≤ 0.05 (Tukey HSD) for the three main factors separately; TS, drilled using a mechanical seeder with rows spaced 20 cm apart without film mulching; FMR&F, film-mulched ridge (an arc with 40 cm wide base and 10 cm height) and furrow (rows spaced 15 cm) sowing method by using an all-in-one machine; WR&NF, wide ridge (25 cm wide base and 12 cm height) and narrow furrow (depth 8 cm, sown into the top-edges of the furrow, rows spaced 12 cm) sowing method by using an all-in-one machine; CK, control (fallow land).*

**FIGURE 1:**
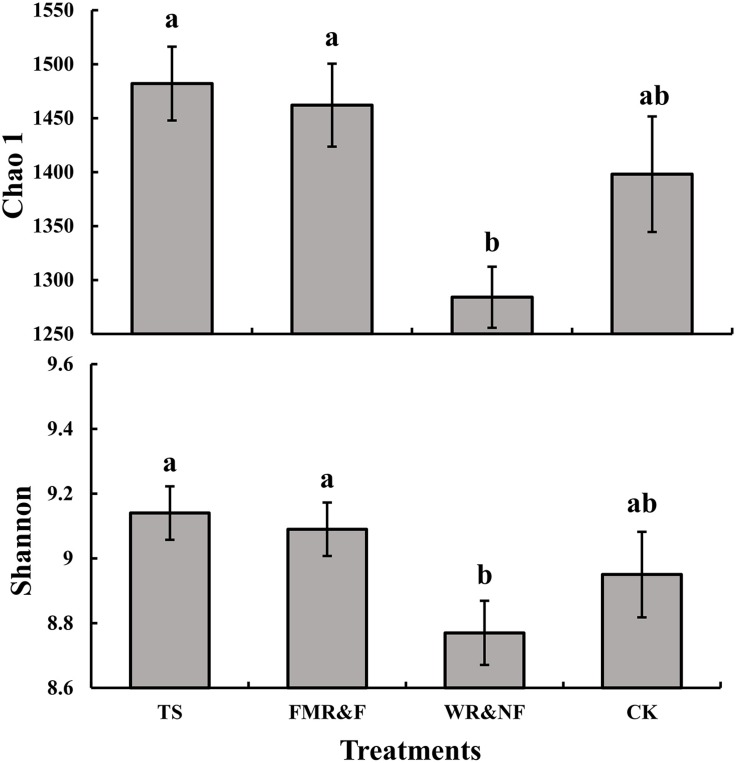
Soil bacterial diversity indices (Chao1 and Shannon) as influenced by different sowing methods. TS = drilled using a mechanical seeder with rows spaced 20 cm apart without film mulching; FMR&F, film-mulched ridge (an arc with 40 cm wide base and 10 cm height) and furrow (rows spaced 15 cm) sowing method by using an all-in-one machine; WR&NF, wide ridge (25 cm wide base and 12 cm height) and narrow furrow (depth 8 cm, sown into the top-edges of the furrow, rows spaced 12 cm) sowing method by using an all-in-one machine; CK, control (fallow land). Chao 1, soil bacterial community richness index; Shannon, soil bacterial community diversity estimator. a, b Means are significantly different at *p* ≤ 0.05 (Tukey HSD).

**FIGURE 2:**
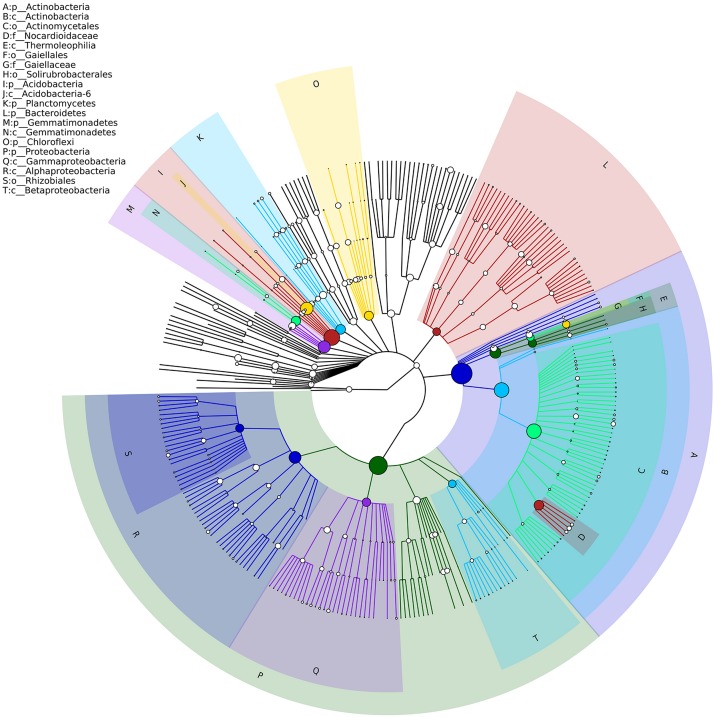
Hierarchy tree graph of samples based on GraPhlAn. The node size corresponds to the average relative abundance of the taxon in all soil samples. The top-ranked 20 most abundant taxa were identified by the alphabet (the shadow color of alphabet corresponds to the nodes color of taxon) and were shown on the upper left. Seven phyla had relative abundance >2%, and almost 97% of soil bacterial sequences were attributed to these seven phyla.

**FIGURE 3:**
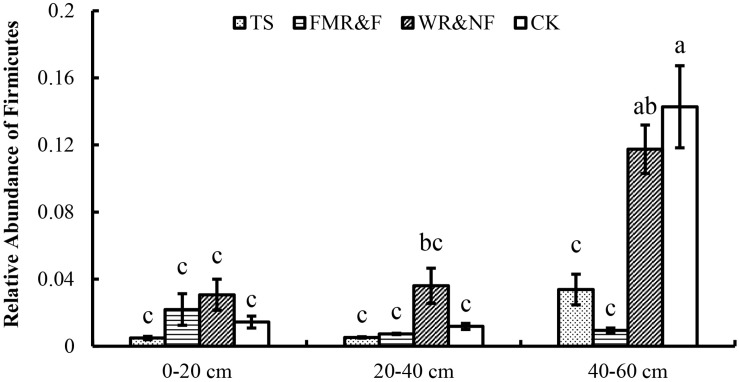
Relative abundance of Firmicutes in soil as influenced by the interaction between sowing method and soil layers. TS = drilled using a mechanical seeder with rows space 20 cm apart without film mulching; FMR&F, film-mulched ridge (a circular arc with 40 cm wide base and 10 cm height) and furrow (row spaced 15 cm)sowing method by using an all-in-one machine; WR&NF, wide ridge (25 cm wide base and 12 cm height) and narrow furrow (depth 8 cm, sown into wide-ward furrow, rows space 12 cm) sowing method by using an all-in-one machine; CK, control (fallow land). a, b, c Means are significantly different at *p* ≤ 0.05 (Tukey HSD).

Soil bacterial α-diversity (Chao1 and Shannon indices) were influenced significantly by the sowing method treatment (Figure 1). The wide ridge and narrow furrow (WR&NF) treatment had significantly lower in bacterial diversity than the TS and FMR&F treatment that did not difference between themselves. Top 20 dominant taxa (ranked based on abundance) were identified by a hierarchy tree graph. Almost 97% of soil bacterial sequences was attributed to seven phyla, including *Actinobacteria, Acidobacteria, Planctomycetes, Bacteroidetes, Gemmatimonadetes, Chloroflexi*, and *Proteobacteria* (Figure 2). The phyla of *Gemmatimonadetes, Planctomycetes, Firmicutes*, and *Verrucomicrobia* differed significantly in the various sowing method treatments (Table 2). The TS treatment had higher abundance of *Gemmatimonadetes* than the FMR&F and WR&NF treatments, and higher abundance of *Planctomycetes* and *Verrucomicrobia* than the WR&NF treatment. On the other hand, the abundance of *Firmicutes* was higher in the WR&NF and control treatments compared to the other sowing treatments, and was influenced significantly by the interaction of sowing method and soil layers treatments (Figure 3).

**TABLE 2:** Relative abundance of dominant phyla in soil as influenced by sowing method, sampling site and soil layers (1-way ANOVA).

Dominant phyla	Treatments								
	Sowing methods			Sampling sites			Soil layers		
	TS	FMR&F	WR&NF	CK	Rhizosphere	Non-rhizosphere	0–20 cm	20–40 cm	40–60 cm
*Acidobacteria*	0.16	0.16	0.15	0.18	0.13b	0.17*a*	0.16	0.16	0.15
*Gemmatimonadetes*	0.11a	0.09b	0.08b	0.09ab	0.09	0.09	0.09	0.09	0.10
*Planctomycetes*	0.07a	0.06ab	0.05b	0.06ab	0.06	0.06	0.07	0.06	0.05
*Chloroflexi*	0.05	0.06	0.05	0.05	0.05b	0.06a	0.05	0.05	0.05
*Firmicutes*	0.02b	0.01b	0.06a	0.06a	0.03	0.03	0.02b	0.02b	0.07a
*Nitrospirae*	0.03	0.03	0.03	0.03	0.03	0.03	0.02b	0.03b	0.04a
*Verrucomicrobia*	0.03a	0.03ab	0.03b	0.02b	0.03	0.03	0.04a	0.03a	0.02b

*a, b Means are different at *p* ≤ 0.05 (Tukey HSD) for the three main factors separately; TS, drilled using a mechanical seeder with rows spaced 20 cm apart without film mulching; FMR&F, film-mulched ridge (an arc with 40 cm wide base and 10 cm height) and furrow (rows spaced 15 cm) sowing method by using an all-in-one machine; WR&NF, wide ridge (25 cm wide base and 12 cm height) and narrow furrow (depth 8 cm, sown into the top-edges of the furrow, rows spaced 12 cm) sowing method by using an all-in-one machine; CK, control (fallow land).*

**FIGURE 4:**
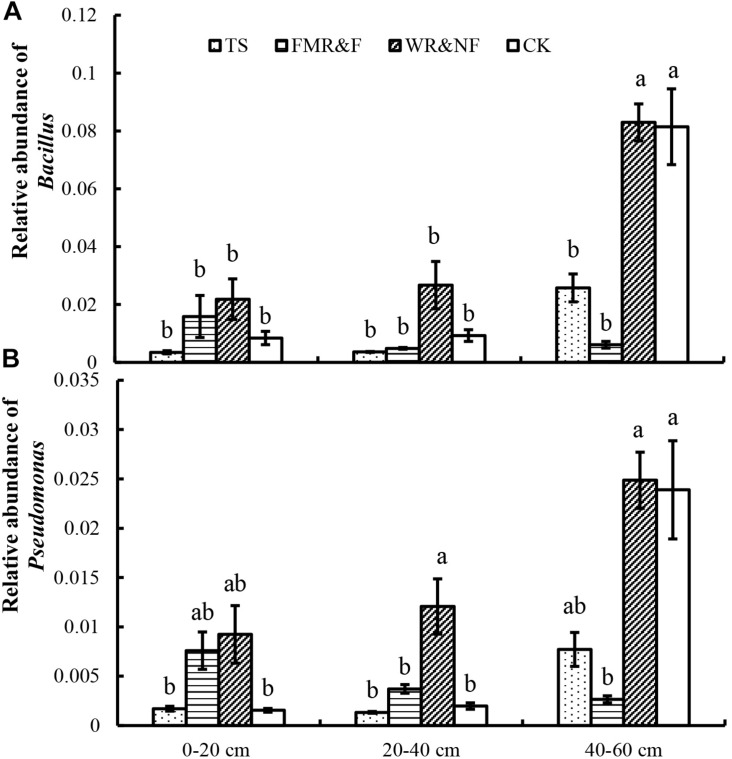
Relative abundance of *Bacillus* **(A)** and *Pseudomonas* **(B)** in soil as influenced by sowing method and soil layers (2-way ANOVA). TS = drilled using a mechanical seeder with rows space 20 cm apart without film mulching; FMR&F, film-mulched ridge (a circular arc with 40 cm wide base and 10 cm height) and furrow (row spaced 15 cm)sowing method by using an all-in-one machine; WR&NF, wide ridge (25 cm wide base and 12 cm height) and narrow furrow (depth 8 cm, sown into wide-ward furrow, rows space 12 cm) sowing method by using an all-in-one machine; CK, control (fallow land). a, b, c Means are significantly different at *p* ≤ 0.05 (Tukey HSD).

The genera of *Bacillus, Streptacidiphilus, Kribbella, Bradyrhizobium, Pseudomonas, Aeromicrobium*, and *Gemmata* differed significantly in the various sowing method treatments (Table 3). The WR&NF treatment had higher abundance of *Bacillus* (phylum *Firmicutes*), *Streptacidiphilus* (phylum *Actinobacteria*), *Bradyrhizobium* (phylum *Proteobacteria*), and *Pseudomonas* (phylum *Proteobacteria*) than the TS treatments. In contrast, the TS treatment had higher abundance of *Kribbella* (phylum *Actinobacteria*), *Aeromicrobium* (phylum *Actinobacteria*), and *Gemmata* (phylum *Planctomycetes*) than the WR&NF treatment. The FMR&F treatment had higher abundance of *Aeromicrobium* (phylum *Actinobacteria*) and lower abundance of *Bradyrhizobium* than the WR&NF treatment, and lower abundance of *Gemmata* than the TS treatments. Abundance of *Bacillus* (phylum *Firmicutes*) and *Pseudomonas* (phylum *Proteobacteria*) were influenced significantly by the interaction of sowing method treatments and soil layers. Abundance of *Bacillus* and *Pseudomonas* genera was higher in the 40–60 cm soil layer in the WR&NF and control treatment compared with the other sowing treatments, but no difference was found among the sowing treatments in the other soil layers (Figure 4). These results revealed that the sowing method treatments influence the properties and bacterial community composition of soil. The WR&NF treatment showed higher abundances of *Bacillus* (phylum *Firmicutes*), *Bradyrhizobium* (phylum *Proteobacteria*) and *Pseudomonas* (phylum *Proteobacteria*) than the TS and FMR&F treatments.

**TABLE 3:** Relative abundance of dominant genera in soil as influenced by sowing method, sampling site and soil layers (1-way ANOVA).

Dominant genera (×10^–^^3^)	Treatments								
	Sowing methods			Sampling sites			Soil layers		
	TS	FMR&F	WR&NF	CK	Rhizosphere	Non-rhizosphere	0–20 cm	20–40 cm	40–60 cm
*Bacillus*	10.95 bc	8.94 c	43.83 a	33.05 ab	22.63	23.15	12.98 b	11.38 b	44.43 a
*Streptacidiphilus*	9.27 b	14.42 ab	18.02 a	9.65 b	14.89	12.1	10.11 b	12.47 ab	17.32 a
*Kribbella*	16.30 a	12.37 ab	12.07 b	7.74 b	14.49	11.44	13.76 a	15.07 a	9.41 b
*Bradyrhizobium*	8.09 b	6.76 b	13.14 a	10.77 ab	10.58	8.76	8.78 ab	8.12 b	11.71 a
*Pseudomonas*	3.59 b	4.66 b	15.39 a	9.14 ab	8.98	7.37	5.52 b	5.18 b	13.48 a
*Kaistobacter*	8.46	7.94	7.09	7.74	7.15	8.32	10.09 a	8.09 a	5.28 b
*Aeromicrobium*	8.58 a	9.09 a	4.90 b	3.53 b	7.16	6.80	8.43 a	8.06 a	4.36 b
*Gemmata*	7.95 a	5.23 b	3.91 b	5.71 ab	5.72	5.68	6.77	5.83	4.50
*Lentzea*	5.22	4.38	6.01	4.57	6.01 a	4.44 b	6.09 a	5.20 ab	4.05 b

*a, b, c Means are different at *p* ≤ 0.05 (Tukey HSD) for the three main factors separately; TS, drilled using a mechanical seeder with rows spaced 20 cm apart without film mulching; FMR&F, film-mulched ridge (an arc with 40 cm wide base and 10 cm height) and furrow (rows spaced 15 cm) sowing method by using an all-in-one machine; WR&NF, wide ridge (25 cm wide base and 12 cm height) and narrow furrow (depth 8 cm, sown into the top-edges of the furrow, rows spaced 12 cm) sowing method by using an all-in-one machine; CK, control (fallow land).*

### Influence of Distance to Roots on Soil Properties and Bacterial Community Composition

Soil properties between rhizosphere and non-rhizosphere were differed significantly. The non-rhizosphere soil showed higher values for all soil properties, except soil moisture, compared to the rhizosphere soil (Table 1). Soil moisture content was higher in the rhizosphere than non-rhizosphere soil, and was influenced significantly by interaction between sampling site and the soil layers treatments (Supplementary Figure S3). Meanwhile, we found that the non-rhizosphere soil had higher abundance of *Acidobacteria* and *Chloroflexi* phyla than the rhizosphere soil (Table 2). The rhizosphere soil had higher abundance of *Lentzea* (phylum *Actinobacteria*) than the non-rhizosphere soil (Table 3).

### Influence of Soil Depth on Soil Properties and Bacterial Community Composition

Soil properties (organic matter, available P and pH etc.) were decreased down the soil profile and significantly higher in the top-soil layer (Table 1). Available N and available K were influenced significantly by the interaction among sowing method, sampling site and soil layer (Supplementary Table S1). High available N and available K concentrations were recorded in the top-soil of non-rhizosphere environment in the WR&NF treatment. Soil moisture content was decreased significantly down the soil profile (Supplementary Figure S3).

Soil bacterial diversity was influenced by the interaction of sowing method and soil depth. The lowest soil bacterial diversity (Chao1 index) was recorded in the 40–60 cm soil layer of the WR&NF treatment (Supplementary Table S3). Soil depth factor strongly influence abundance of *Firmicutes, Nitrospirae*, and *Verrucomicrobia* (Table 2). The 40–60 cm soil layer had higher abundance of *Firmicutes* and *Nitrospirae* than the other two soil layers. In contrast, the lowest abundance of *Verrucomicrobia* was recorded in the 40–60 cm soil layer. Soil depth strongly influenced abundance of *Bacillus, Streptacidiphilus, Kribbella, Bradyrhizobium, Pseudomonas, Kaistobacter, Aeromicrobium*, and *Lentzea* (Table 3). The top soil layer had higher abundance of *Kribbella, Kaistobacter, Aeromicrobium* and *Lentzea* than the 40–60 cm soil layer. In contrast, the lowest abundance of *Bacillus* (phylum *Firmicutes*),*Streptacidiphilus* (phylum *Actinobacteria*) and *Pseudomonas* (phylum *Proteobacteria*) were recorded in the top-soil. The highest abundance of *Kribbella* (phylum *Actinobacteria*) was recorded in the 20–40 cm soil layer. Hence, soil depth appeared to be a strong factor that influenced soil properties and bacterial community composition. Soil properties were significantly decreased down the soil profile.

### Correlation of Soil Properties and Bacterial Community Composition

We found that all the six soil properties tested were significantly (*p* ≤ 0.05) and positively correlated with each other (Supplementary Table S2). Also, soil bacterial diversity correlated positively (*p* ≤ 0.05) with available N (*p* = 0.02), P (*p* = 0.03), K (*p* = 0.04), pH (*p* = 0.05), and soil moisture contents (*p* = 0.01), but not organic matter (*p* = 0.17). Spearman’s rank order correlation analysis showed strong correlation among dominant taxa, treatments and soil properties (Supplementary Table S4). Abundance of *Firmicutes* and *Nitrospirae* phyla was significantly negatively correlated, and that of *Verrucomicrobia* positively correlated, with the soil properties except soil pH. Abundance of *Proteobacteria* was significantly positively correlated with SOM (*p* < 0.01) and soil moisture content (*p* < 0.01). Abundance of *Planctomycetes* phylum was positively correlated with available N (*p* = 0.02), and abundance of *Bacteroidetes* had a positive correlation with (*p* = 0.03) the soil moisture content.

Among genera (Supplementary Table S4), abundance of *Bacillus* was significantly negatively correlated with the soil properties except soil pH (*p* = 0.16). Abundance of *Kaistobacter* had a strong positive correlation with available N, P, K, organic matter and soil moisture content. Abundance of *Streptacidiphilus* was significantly negatively correlated with available P and available N, K and organic matter. Abundance of *Bradyhizobium* had a significant negative correlation with soil pH (*p* = 0.04) and soil moisture content (*p* = 0.04). Abundance of *Pseudomonas* was significantly negatively correlated with available N, P, and K. Abundance of *Aeromicrobium* was significantly positively correlated with SOM, available N and soil moisture content as well as available P and K. Abundance of *Gemmata* and *Lentzea* was strongly positively correlated with available N (*p* = 0.01) and SOM (*p* = 0.04), whereas abundance of *Nocardioides* had a significant negative correlation (*p* = 0.02) with available P.

## Discussion

Sowing methods that include ridging/furrowing are likely to alter physicochemical soil properties as well as soil bacterial diversity and community structure (Shah et al., 2013). The aim of this study was to investigate the response of soil bacterial diversity and abundance to different sowing method treatments, which would alter the abundance of soil beneficial bacteria that contribute to the improvement of soil qualities in winter wheat-summer maize crop rotation system on the Loess Plateau. We found the correlations between the soil bacterial diversity and abundance of predominant taxa and selected soil properties.

### Influence of Sowing Methods on Soil Properties and Bacterial Community Composition

We found that the FMR&F treatment increased SOM and available P compared with the WR&NF and TS treatments, which did not differ between themselves (Table 1). It has recently been suggested that plastic film mulching would increase soil organic matter content (SOM) via increasing local soil environment temperature and decreasing periodic physical disturbance (Balesdent et al., 2000; Liu et al., 2009; Zhang et al., 2011). In addition, plastic film mulching provided a stable soil micro-environment that enhanced the nutrient cycling in top soil layers and prevented water loss (Li et al., 2004; Anikwe et al., 2007; Mulumba and Lal, 2008). In the present study, the WR&NF treatment was a conservation sowing technique with no-deep tillage (Zhang et al., 2012). Nutrient distribution and potential N mineralization in soil were affected by different tillage practices (Salinas-Garcìa et al., 2002). The highest available N and available K were recorded in the top-soil of the WR&NF treatment (Supplementary Table S1). In addition, soil chemical characteristics (e.g., soil available N, available K, and pH) were changed under different tillage intensities (López-Fando and Pardo, 2009). Accordingly, the WR&NF sowing method with minimum tillage improved the nitrogen cycling in soil compared to the other treatments.

Soil microbial diversity is an important indicator of soil quality (Anderson, 2003), influenced by tillage (Lupwayi et al., 1998) and cropping system (Moore et al., 2000). Tillage practice may lead to a decrease in bacterial community diversity due to homogenization of soil and disturbing the unique micro-environment microorganisms inhabitant (Sengupta and Dick, 2015). One interesting finding was that the WR&NF treatment, a sowing method with no-deep tillage, reduced the soil bacterial diversity compared to the TS and FMR&F treatments (Figure 1). The lowest bacterial diversity (Chao 1 index) was recorded in the deeper soil layers of the WR&NF treatment (Supplementary Table S3). This is likely a consequence of the narrow furrow (8 cm depth) in top-soil layers of WR&NF treatment. Creating furrows for seed and fertilizer placement, as often used in no-till farming, would lead to soil disturbance in the top-soil layers (Barr et al., 2016), resulting in changed soil properties (Zhou et al., 2012) and soil microbial community structure (Qin et al., 2017).

Soil bacterial community structure and composition were influenced strongly by soil physicochemical properties and environmental variables (Arroyo et al., 2015). Our results indicated that abundance of predominant taxa showed significant difference among the different sowing method treatments. Abundance of *Gemmatimonadetes, Verrucomicrobia* and *Planctomycetes* phyla that are indicative of the soil nutrient status and soil carbon quality (Cederlund et al., 2014) did not differ between the FMR&F and the WR&NF treatments, but were significantly lower than the TS treatment (Tables 2). In particular, the phylum *Firmicutes*, with many anaerobic representatives (Teixeira et al., 2010), was higher in the 40–60 cm soil layer in the WR&NF treatment compared with the other treatments (Figure 3). The WR&NF treatment had recruited more beneficial bacteria than TS treatment (Table 3 and Figure 4), such as *Streptacidiphilus* (phylum Actinobacteria), *Pseudomonas* (phylum Proteobacteria) (Gügi et al., 1991), *Bacillus* (phylum Firmicutes) (Skraly and Cameron, 1998), and *Bradyrhizobium* (phylum Proteobacteria) (Wu et al., 2014). Also, the sowing methods treatments were strongly influenced the abundance of *Streptacidiphilus, Bradyrhizobium, Bacillus*, and *Pseudomonas* (Supplementary Table S4). Hence, we can conclude that different sowing treatments changed soil bacterial diversity and abundance as well as soil properties. These results are consistent with some previous studies in which soil bacterial community structure and composition were correlated with SOM and nutrient contents (Ansola et al., 2014; Hartmann et al., 2015; Nannipieri et al., 2017), soil moisture and plant interaction (Chen et al., 2007), soil pH (Fierer and Jackson, 2006; Lauber et al., 2009; Kuramae et al., 2012), different farming systems (Esperschuetz et al., 2007), and different tillage practices (Sengupta and Dick, 2015).

Previous reports showed that the no-tillage treatment contributed to increased yield (Soomro et al., 2009), spike length and number of grains per spike (Tanveer et al., 2003), drought-resistance and conservation of soil moisture (Christian et al., 2005). Similarly, the WR&NF treatment, as a no-mulching film and no-deep tillage sowing technique in this study, altered soil bacterial community composition and increased soil beneficial bacteria abundance. Annually, large amounts of plastic films used for agricultural mulching contaminate the soil environment in China (Chen et al., 2013). In contrast, the WR&NF treatment avoided plastic contamination of soils (Sun et al., 2015). Moreover, the WR&NF treatment had higher wheat yield than the TS and FMR&F treatment in latter experiments. Based on the results presented here, we suggest that the WR&NF treatment was the most suitable sowing technique in winter wheat-summer maize rotation on Loess Plateau.

### Influence of Distance to Roots on Soil Properties and Bacterial Community Composition

Some reports showed that root nutrient uptake, exudation and microbial activity led to variation in soil properties between the rhizosphere and non-rhizosphere soils (Marschner et al., 2001; Raynaud, 2010). In our study, we found that compared to rhizosphere soil, the non-rhizosphere soil had higher SOM, available P, pH. and soil moisture content (Table 1 and Supplementary Figure S3), reflecting relatively high uptake of P and water by roots that also acidified the rhizosphere soil environment (Russell, 1979; Pierret et al., 2007).

We found a plant-dependent rhizosphere effect in soil bacterial community composition (Smalla et al., 2001; Uroz et al., 2010). Abundance of *Acidobacteria* and *Chloroflexi* phyla was significantly higher in the non-rhizosphere than rhizosphere soil, whereas *Lentza* genus was more abundant in the rhizosphere (Tables 2, 3). Saleem et al. (2018) suggested that root system architecture altered the root microbiome structure. Marschner and Timonen (2005) demonstrated that an interaction between plant species and mycorrhizal colonization affected soil bacterial community structure in the rhizosphere. Also, soil type, nutrition, fertilization and root zone location would influence soil bacterial diversity (Marschner et al., 2001, 2004; Berg and Smalla, 2009).

Fierer and Jackson (2006) reported that soil properties determined soil bacterial diversity and richness. We also found that abundance of *Lentza* genus was correlated positively with SOM and available N (Tables 3 and Supplementary Table S4). We suggest that soil properties influence the bacterial community composition by providing various nutritional environments for bacterial growth and reproduction. In other studies, root system size and distribution loosened soil structure and improved microclimate, impacting soil bacterial community structure (Marschner et al., 2004). Root exudation at the root apices also influenced bacterial community in the rhizosphere (Dennis et al., 2010). Understanding the influence of root system and root exudation on the soil bacterial diversity and abundance hinges on additional experiment evidence because soil properties are interdependent and do not respond in the same way to the distance to roots (Ansola et al., 2014).

### Influence of Soil Depth on Soil Properties and Bacterial Community Composition

Salome et al. (2010) reported differences in pedological, environment and physicochemical properties in different soil layers. In the study presented here, we also recorded soil properties differing significantly down the soil profile (Table 1) and correlating negatively (*p* < 0.01) with soil layers (Supplementary Table S4). Kramer and Gleixner (2008) suggested that SOM varied with soil depth because distinct carbon preferences of microbial community. In addition, Jobbágy and Jackson (2001) reported that root systems influenced nutrient contents in different soil layers. Soil available potassium spatial distribution over depth was affected by land management and fertilization (Zhang et al., 2013). Accordingly, we confirmed that soil properties significantly worsened with depth mainly due to decreased nutrition and soil microbial activity.

Fierer et al. (2003) reported that vertical and spatial distribution of bacterial community down the soil profile was influenced by dwindling carbon resources at depth. In the present study, *Verrucomicrobia* phylum and *Kaistobacter* (phylum Proteobacteria), *Aeromicrobium* (phylum Actinobacteria), and *Lentzea* (phylum Actinobacteria) genera that were abundant in the top-soil layer, might have been responsible for specific suppression of the soil-borne plant pathogens (Weller et al., 2002). In contrast, the 40–60 cm soil layer had more *Nitrospirae* and *Firmicutes* phyla and genera *Bacillus* (phylum *Firmicutes*), *Streptacidiphilus* (phylum Actinobacteria), *Bradyrhizobium* (phylum Proteobacteria), and *Pseudomonas* (phylum Proteobacteria) (Tables 2, 3), which were reported to be involved in the soil N-recycling (Kuypers et al., 2018), soil inorganic phosphate solubilization (Rodríguez et al., 2006), soil heterotrophic metabolism (Tang et al., 2011) and cellulose decomposition in soil (Pepe-Ranney et al., 2016). These predominant taxa were correlated (negatively or positively) with the available N, P, K, and SOM as well as some other soil properties (Supplementary Table S4). These results were consistent with the previous reports, in which bacteria abundance was dependent on soil depth, and was correlated significantly with soil properties such as organic carbon content and total nitrogen (Will et al., 2010). Soil pH may impose a micro-environment stress on bacteria and impact the relative abundance of predominant taxa (Lauber et al., 2008). SOM decomposition vs. stabilization may alter bacterial community structure via influences on soil carbon stock (Six et al., 2002; Birkhofer et al., 2012). Therefore, we suggest that soil properties (including SOM, available N, P, and K, etc.) contributed to soil bacterial community differences via the variation in availability of carbon and nutrients in different soil layers. Barrios (2007) revealed that specific soil microorganism could influence directly the crop yield and could impact indirectly the crop productivity via influencing the soil carbon and nutrient cycling and soil structure modification. Symbiotic nitrogen-fixing bacteria (e.g., *Bradyrhizobium*) and free-living nitrogen-fixing bacteria (e.g., *Pseudomonas*) were attached to the crop roots and efficiently colonized root surface, contributing to sustainable crop growth enhancement and crop productivity (Hayat et al., 2010).

### Co-correlation of Keystone Bacteria Genera

**FIGURE 5:**
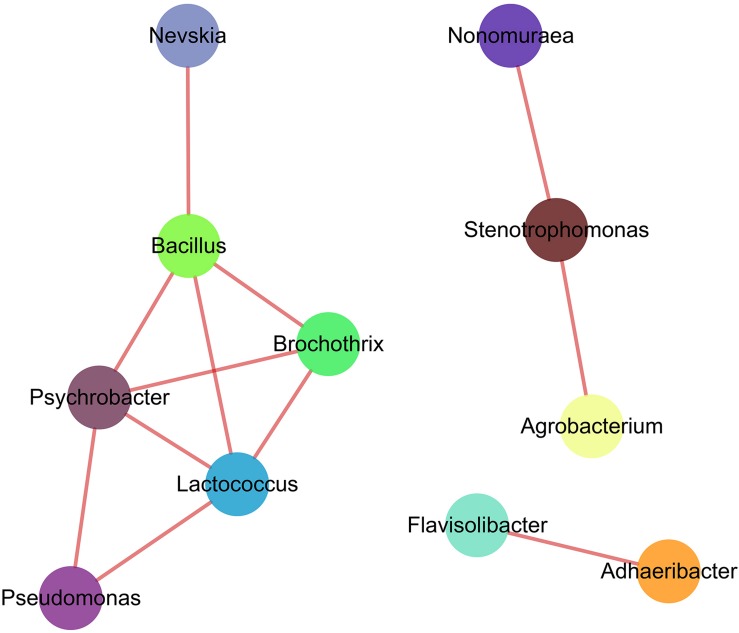
Correlation network graph of predominant genera based on Spearman’s rank correlations. Every node represents the keystone genera. Solid lines between two nodes indicate significant positive correlation (| ρ| > 0.8 and *p* < 0.01).

Soil bacteria participate in the vital biological and ecosystem functions, being a principal driving force for example in soil carbon, nitrogen and phosphorus cycling. Rampelotto et al. (2015) revealed that interactions among different bacterial taxa resulted in a unique pattern of clustered topology. Co-occurrence and interactions among soil bacterial species not only defined the species characteristics, but also drive the ecosystem functionality of bacterial community (Fuhrman, 2009). In the present study, we showed that, among top 50 dominant genera (Figure 5), 11 genera had positive co-correlations (| ρ| > 0.8), with *Bacillus* (Skraly and Cameron, 1998), *Pseudomonas* (Gügi et al., 1991*), Nevskia* and *Lactococcus* being the keystone genera in the soil bacterial community based on the higher abundance in soil samples and the network topology analyses (Barberán et al., 2012; Lupatini et al., 2014). Co-occurrence of soil bacteria was affected by several ecological processes, including competition and habitat filtering (Woodcock et al., 2007). Ma et al. (2016) reported that co-occurrence and interactions among bacterial taxa contributed to soil ecosystem functions and species diversity. Soil bacterial species correlated with soil properties and played a critical role in soil biology functions via potential interactions and habitat-sharing effects (Cardinale et al., 2015; Menezes et al., 2015). We concluded that variations in soil properties and associations among keystone taxa may jointly influence or reflect the feedback from the soil bacterial community composition in the winter wheat-summer maize rotation on the Loess Plateau (King et al., 2012).

## Conclusion

Soil bacterial diversity and community composition were influenced by the different sowing method treatments. The WR&NF sowing method significantly decreased the bacterial diversity compared to the TS and FMR&F treatments. However, the beneficial bacterial taxa, such as genera *Bacillus* and *Pseudomonas*, were abundant in the WR&NF sowing method treatment. They showed a stronger correlation with soil properties and contributed to the soil nutrient cycling and crop productivity. Therefore, we suggested that the WR&NF treatment, as a no-mulching film and no-deep tillage sowing method, would be the most suitable sowing technique in the winter wheat-summer maize rotation on the Loess Soil.

## Data Availability Statement

The study accession number is SRP231783, the bioproject accesion number is PRJNA590553, the biosample accession number is SAMN13335931.

## Author Contributions

ZY and CH conceived and designed the experiment. CH and XH prepared the experimental materials, carried out the experiment and collected and analyzed data. CH wrote the first draft of the manuscript. ZY, YC, and ZR revised the manuscript. ZY and YC addressed authors’ responses to comments. ZR edited language. All authors approved the final version of the manuscript for publication.

## Conflict of Interest

The authors declare that the research was conducted in the absence of any commercial or financial relationships that could be construed as a potential conflict of interest.

## Footnotes

1 http://qiime.org/

2 http://ccb.jhu.edu/software/FLASH/

3 http://www.mothur.org/
