# Supplementary material for: Sowing Methods Influence Soil Bacterial Diversity and Community Composition in a Winter Wheat-Summer Maize Rotation System on the Loess Plateau
Source: Front Microbiol. 2020 Feb 18;11:192. doi: 10.3389/fmicb.2020.00192 (PMC7040079; doi:10.3389/fmicb.2020.00192)
Supplement: Supplementary file 1 [file Data_Sheet_1.docx]

*Supplementary materials*

Table S1 Physicochemical soil properties among sowing methods, sampling sites and soil layers (analysed by orthogonal contrasts)

| Sowing methods | Sampling sites | Soil layers | Organic matter (g/kg) | Available P (mg/kg) | pH_water_ | Soil moisture  (% w/w) | Available N  (mg/kg) | Available K  (mg/kg) |
| --- | --- | --- | --- | --- | --- | --- | --- | --- |
| TS | rhizosphere | 0-20 cm | 12.24 | 10.65 | 7.15 | 34.45 | 73 de | 129 c |
| TS | rhizosphere | 20-40 cm | 7.16 | 4.45 | 7.06 | 31.41 | 51 fghijk | 89 hi |
| TS | rhizosphere | 40-60 cm | 5.59 | 2.81 | 7.04 | 29.06 | 64 ef | 86 hi |
| TS | non-rhizosphere | 0-20 cm | 13.46 | 11.60 | 7.21 | 31.21 | 108 ab | 135 b |
| TS | non-rhizosphere | 20-40 cm | 8.40 | 6.14 | 7.13 | 29.31 | 52 fghij | 97 fg |
| TS | non-rhizosphere | 40-60 cm | 6.45 | 3.45 | 7.10 | 23.30 | 33 k | 85 i |
| FMR＆F | rhizosphere | 0-20 cm | 16.70 | 12.42 | 7.16 | 41.93 | 87 cd | 139 ab |
| FMR＆F | rhizosphere | 20-40 cm | 9.41 | 6.30 | 6.97 | 37.31 | 58 efg | 92 gh |
| FMR＆F | rhizosphere | 40-60 cm | 6.41 | 5.63 | 6.84 | 35.49 | 47 fghijk | 74 j |
| FMR＆F | non-rhizosphere | 0-20 cm | 17.38 | 12.66 | 7.44 | 39.57 | 93 bc | 113 e |
| FMR＆F | non-rhizosphere | 20-40 cm | 9.62 | 6.95 | 7.16 | 35.11 | 55 efghi | 100 f |
| FMR＆F | non-rhizosphere | 40-60 cm | 7.62 | 4.75 | 7.08 | 33.55 | 41 ghijk | 98 f |
| WR＆NF | rhizosphere | 0-20 cm | 13.16 | 11.41 | 6.98 | 36.37 | 72 de | 121 d |
| WR＆NF | rhizosphere | 20-40 cm | 8.23 | 5.48 | 7.17 | 32.48 | 49 fghijk | 86 hi |
| WR＆NF | rhizosphere | 40-60 cm | 6.54 | 3.63 | 7.09 | 28.55 | 37 ijk | 75 j |
| WR＆NF | non-rhizosphere | 0-20 cm | 12.63 | 12.55 | 7.15 | 34.61 | 112 a | 142 a |
| WR＆NF | non-rhizosphere | 20-40 cm | 8.44 | 6.27 | 7.00 | 29.48 | 57 efgh | 101 f |
| WR＆NF | non-rhizosphere | 40-60 cm | 7.11 | 3.83 | 7.15 | 22.70 | 39 hijk | 90 hi |
| CK | non-rhizosphere | 0-20 cm | 11.28 | 12.54 | 7.13 | 33.83 | 112 a | 124 cd |
| CK | non-rhizosphere | 20-40 cm | 9.22 | 6.21 | 7.09 | 28.21 | 51 fghijk | 101 f |
| CK | non-rhizosphere | 40-60 cm | 8.42 | 4.21 | 6.82 | 21.31 | 34 jk | 90 hi |

Means followed by different letters in a column are significantly different at *P*≤0.05 (Tukey HSD).

TS = drilled using a mechanical seeder with rows spaced 20 cm apart without film mulching;

FMR＆F = film-mulched ridge (an arc with 40 cm wide base and 10 cm height) and furrow (rows spaced 15 cm) sowing method by using an all-in-one machine;

WR＆NF = wide ridge (25 cm wide base and 12 cm height) and narrow furrow (depth 8 cm, sown into the top-edges of the furrow, rows spaced 12 cm) sowing method by using an all-in-one machine;

CK = control (fallow land)

Table S2 Pearson correlation coefficients between physicochemical soil properties and bacterial diversity indices

| Variables | Available N | Available P | Available K | pH_water_ | Soil moisture (_w/w_) | Chao 1 | Shannon |
| --- | --- | --- | --- | --- | --- | --- | --- |
| Organic matter | 0.74** | 0.90** | 0.82** | 0.40** | 0.66** | 0.12 | 0.17 |
| Available N |  | 0.87** | 0.84** | 0.38** | 0.55** | 0.29* | 0.32* |
| Available P |  |  | 0.89** | 0.38** | 0.67** | 0.22 | 0.27* |
| Available K |  |  |  | 0.31* | 0.49** | 0.20 | 0.26* |
| pH_water_ |  |  |  |  | 0.25* | 0.21 | 0.25* |
| Soil moisture (_w/w_) |  |  |  |  |  | 0.30* | 0.32** |
| Chao1 |  |  |  |  |  |  | 0.96** |

* significant difference at *p*≤0.05, ** significant difference at *p*≤0.01;

Chao1 = soil bacterial community richness index; Shannon = soil bacterial community diversity estimator

Table S3 Soil bacterial diversity between sowing methods and soil layers (analysed by orthogonal contrasts)

| Sowing methods | Soil layers | Chao 1 | Shannon |
| --- | --- | --- | --- |
| TS | 0-20 cm | 1496 a | 9.24 a |
| TS | 20-40 cm | 1563 a | 9.29 a |
| TS | 40-60 cm | 1385 abc | 8.89 abc |
| FMR＆F | 0-20 cm | 1362 abc | 8.91 abc |
| FMR＆F | 20-40 cm | 1550 a | 9.28 a |
| FMR＆F | 40-60 cm | 1474 a | 9.06 a |
| WR＆NF | 0-20 cm | 1391 ab | 9.04 a |
| WR＆NF | 20-40 cm | 1380 abc | 8.95 ab |
| WR＆NF | 40-60 cm | 1080 c | 8.32 bc |
| CK | 0-20 cm | 1534 a | 9.25 a |
| CK | 20-40 cm | 1542 a | 9.34 a |
| CK | 40-60 cm | 1118 bc | 8.25 c |

Means followed by different letters in a column are significantly different at *P*≤0.05 (Tukey HSD).

TS = drilled using a mechanical seeder with rows spaced 20 cm apart without film mulching;

FMR＆F = film-mulched ridge (an arc with 40 cm wide base and 10 cm height) and furrow (rows spaced 15 cm) sowing method by using an all-in-one machine;

WR＆NF = wide ridge (25 cm wide base and 12 cm height) and narrow furrow (depth 8 cm, sown into the top-edges of the furrow, rows spaced 12 cm) sowing method by using an all-in-one machine;

CK = control (fallow land)

Chao 1 = soil bacterial community richness index; Shannon = soil bacterial community diversity estimator

Table S4 Spearman’s rank correlation among predominant taxa, treatments and physicochemical soil properties

| Dominant taxa and treatments | Physicochemical soil properties and treatments | | | | | | | | |
| --- | --- | --- | --- | --- | --- | --- | --- | --- | --- |
|  | **Organic matter** | **Available N** | **Available P** | **Available K** | **pH_water_** | **Soil moisture** | **Sowing methods** | **Sampling sites** | **Soil layers** |
| Phyla | | | | | | |  |  |  |
| *Actinobacteria* | -0.01 | -0.03 | -0.08 | -0.07 | -0.04 | 0.17 | -0.09 | -0.22 | -0.04 |
| *Proteobacteria* | 0.38** | 0.28* | 0.32* | 0.24 | 0.06 | 0.40** | -0.04 | -0.16 | -0.31 |
| *Acidobacteria* | 0.04 | 0.13 | 0.18 | 0.10 | 0.16 | -0.09 | 0.07 | 0.45** | -0.05 |
| *Gemmatimonadetes* | -0.01 | 0.08 | 0.06 | 0.05 | -0.10 | -0.04 | -0.25* | 0.01 | -0.07 |
| *Planctomycetes* | 0.06 | 0.30* | 0.21 | 0.17 | 0.20 | 0.12 | -0.30* | 0.14 | -0.17 |
| *Chloroflexi* | 0.22 | 0.11 | 0.22 | 0.17 | 0.16 | 0.10 | 0.22 | 0.38** | -0.17 |
| *Bacteroidetes* | 0.08 | 0.15 | 0.04 | 0.06 | 0.06 | 0.28* | -0.24 | -0.29 | -0.12 |
| *Nitrospirae* | -0.44** | -0.39** | -0.29* | -0.39** | -0.11 | -0.30* | 0.15 | 0.23 | 0.49** |
| *Firmicutes* | -0.38** | -0.44** | -0.44** | -0.34** | -0.18 | -0.47** | 0.42** | 0.06 | 0.49** |
| *Verrucomicrobia* | 0.40** | 0.39** | 0.42** | 0.38** | 0.23 | 0.45** | -0.33** | -0.16 | -0.48** |
| Genera | | | | | | |  |  |  |
| *Bacillus* | -0.37** | -0.44** | -0.45** | -0.32** | -0.18 | -0.51** | 0.46** | 0.11 | 0.47** |
| *Streptacidiphilus* | -0.29* | -0.28* | -0.34** | -0.30* | -0.08 | -0.14 | 0.29* | -0.16 | 0.33** |
| *Kribbella* | 0.16 | 0.15 | 0.11 | 0.09 | 0.08 | 0.24 | -0.37** | -0.25 | -0.26* |
| *Bradyrhizobium* | -0.21 | -0.21 | -0.21 | -0.17 | -0.26* | -0.26* | 0.31* | -0.25 | 0.25* |
| *Pseudomonas* | -0.23 | -0.37** | -0.33** | -0.28* | -0.21 | -0.23 | 0.35** | -0.17 | 0.36** |
| *Kaistobacter* | 0.48** | 0.40** | 0.40** | 0.45** | 0.15 | 0.30* | -0.17 | 0.10 | -0.47** |
| *Nocardioides* | -0.20 | -0.17 | -0.29* | -0.18 | -0.13 | -0.10 | 0.01 | -0.26 | 0.10 |
| *Aeromicrobium* | 0.32** | 0.37** | 0.27* | 0.31* | 0.22 | 0.41** | -0.48** | -0.07 | -0.40** |
| *Gemmata* | 0.12 | 0.32* | 0.23 | 0.21 | 0.14 | 0.12 | -0.33** | 0.08 | -0.23 |
| *Lentzea* | 0.26* | 0.28* | 0.19 | 0.17 | 0.09 | 0.20 | 0.03 | -0.33* | -0.34** |
| Treatments |  |  |  |  |  |  |  |  |  |
| Sowing methods | 0.11 | -0.05 | 0.12 | 0.08 | -0.15 | -0.12 | 1 | 0.22 | 0.00 |
| Sampling site | 0.14 | 0.03 | 0.13 | 0.28* | 0.22 | -0.34** | 0.22 | 1 | 0.00 |
| Soil layers | -0.88** | -0.88** | -0.90** | -0.86** | -0.36** | -0.62** | 0.00 | 0.00 | 1 |

* significant difference at *p*≤0.05 ** significant difference at *p*≤0.01


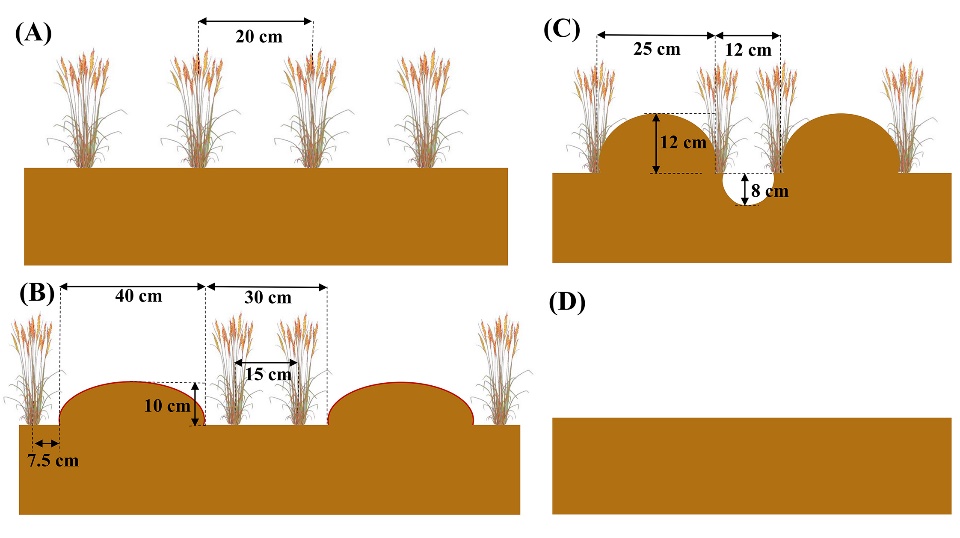


Figure S1 A schematic diagram of different sowing methods. (A) the traditional sowing method (TS), (B) the film-mulched ridge and furrow sowing method (FMR＆F), (C) the wide ridge and narrow furrow sowing method (WR＆NF), (D) the control (fallow land). The red curve in (B) indicated the film mulching


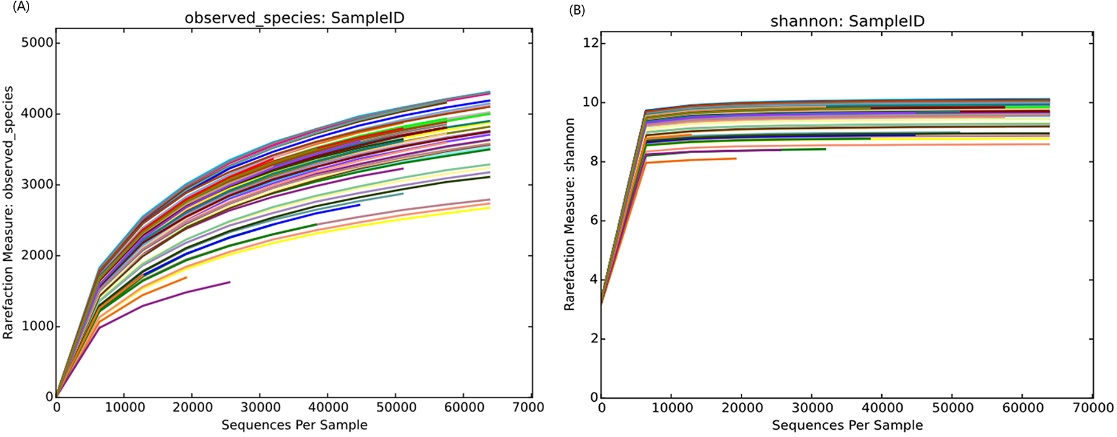


Figure S2 Rarefaction curves of OTUs (A) and the Shannon-Wiener indexes (b) of 16S sequences. The smooth rarefaction curves indicate a good amount of sequencing data. The flat Shannon-Wiener curves indicates that the vast majority of microbes are reflected in the samples


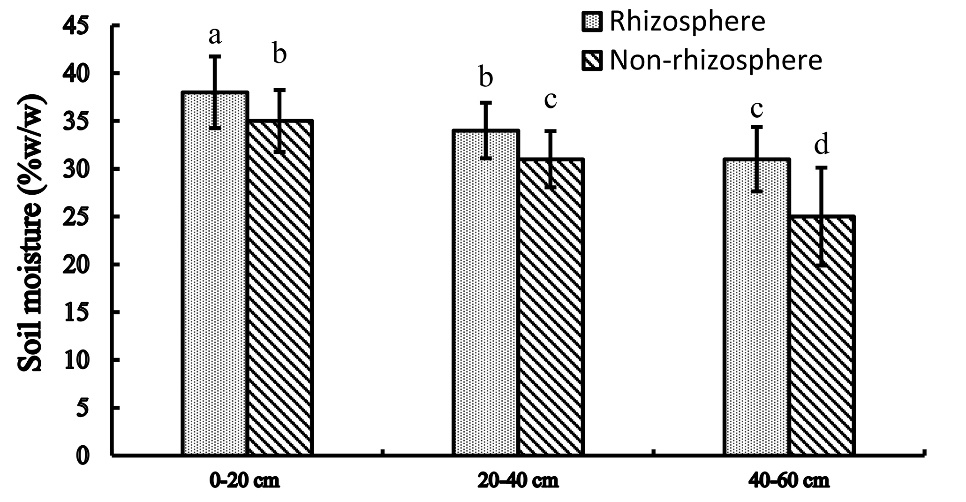


Figure S3 Soil moisture content as influenced by sampling site and soil layers (2-way ANOVA).

a, b, c, d Means are significantly different at *p*≤0.05 (Tukey HSD)
